# Brain metastases as primary manifestation of a melanocytic malignant peripheral nerve sheath tumor in a 60-year-old man

**DOI:** 10.1186/1471-2377-7-2

**Published:** 2007-01-16

**Authors:** Johannes Tilgner, Klaus Müller, Nadir Ghanem, Johannes Lutterbach, Jan Vesper

**Affiliations:** 1 Dept. of Stereotactic Neurosurgery, Universitätsklinik Freiburg, Germany; 2 Dept. of Neuropathology, Universitätsklinik Freiburg, Germany; 3 Dept. of Diagnostic Radiology, Universitätsklinik Freiburg, Germany; 4 Dept. of Radiotherapy, Universitätsklinik Freiburg, Germany

## Abstract

**Background:**

Malignant peripheral nerve sheath tumors are rare tumor entities that originate from peripheral nerve sheaths and have an unfavorable prognosis. Metastatic spread to the cerebral parenchyma is absolutely rare. This case report describes the clinical course in a 60-year-old man whose tumor came to medical attention because of a seizure.

**Case presentation:**

Magnetic resonance imaging demonstrated two intracerebral lesions. The symptomatic lesion was removed microneurosurgically and histology demonstrated a metastasis from a malignant peripheral nerve sheath tumor. Postoperatively, whole-brain irradiation was performed. The primary tumor was identified in the area of the sciatic nerve on the right. Follow-up 14 months after resection showed that there was no progression of the intracerebral lesions but an increase in size and number of distant metastases.

**Conclusion:**

There are no generally accepted guidelines for the treatment of malignant peripheral nerve sheath tumors with cerebral metastases. This case report presents and discusses one possible therapeutic approach. Due to the poor overall prognosis, the least invasive therapy should be chosen.

## Background

The term "malignant peripheral nerve sheath tumor"(MPNST) comprises various entities including malignant schwannoma, neurofibrosarcoma, and neurogenic sarcoma. MPNSTs are rare with an incidence of 1:100,000. About half of the tumors occur in patients with type I neurofibromatosis[1,2]. Interestingly, about 10% of patients have a history of radiotherapy. Reliable data on survival is not available due to the rarity of the tumor and the heterogeneous patient population. One study reports a 5-year survival rate of 64% [3]as compared with 72–78% for sarcomas[4]. The prognosis appears to depend on the tumor size (more unfavorable for tumors >5 cm), the histological grade and the resection margin in patients operated on[2]. There are two different grading classifications: One depends on the World Health Organisation (WHO) classification for soft-tissue tumors with a grading scale from I-III, the other one on the WHO classification for tumors of the nervous system with a grading scale IIIandIV[5,6]. The cell type from which MPNSTs are derived has not been yet identified. According to the WHO definition, MPNSTs are tumors that arise from a peripheral nerve or histologically show nerve sheath differentiation[2]. MPNST is a highly aggressive tumor with a high rate of local recurrence and pulmonary and visceral metastatic spread. Brain metastases are extremely rare and only eleven cases have been published since 1963. We report the case of a 60-year-old man presenting with the initial symptom of a focal seizure and in whom magnetic resonance imaging (MRI) demonstrated two intracerebral lesions.

## Case presentation

### Clinical history

**Figure 1:**
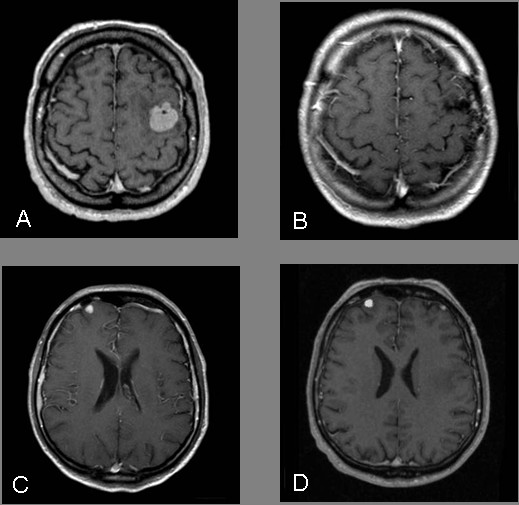
**A**, **C **Pre-Operative MRI: contrast-enhancing lesion in the left and right frontal lobe. **B **MRI follow-up after 6 month: no recurrence of the tumor after resection and irradiation (left frontal). **D **MRI follow-up after 6 month: no progression of the tumor after irradiation only (right frontal).

A 60-year-old man with mental retardation of unclear origin who was otherwise healthy suffered a focal seizure with speech disturbance. Two intracerebral lesions were identified by magnetic resonance imaging (MRI) of the head (Figure 1A+C).

### Physical examination

The patient's neurological status on admission was normal. There were no clues to phakomatosis in the patient's or in the family history. Neither did the clinical findings point in this direction. No further seizures occurred after initiation and starting phenytoin therapy.

### Imaging and laboratory study

Initial MRI demonstrated a contrast-enhancing lesion in the left frontal lobe and a smaller lesion in the right frontal lobe (Figure 1A+C). No other focal lesions were identified. Laboratory tests were normal except for elevations of the following parameters: D-dimer level of 254, neuron-specific enolase level of 13.1, and lambda and kappa light chain levels of 1.63 and 4.64, respectively. The CSF cell count and protein content were normal.

### Patient treatment

Since the lesions had no mass effect, initially a stereotactic biopsy was obtained from the left frontal lesion. Histologically, a semimalignant melanocytoma with the differential diagnosis of a MPNST was diagnosed. The lesion was then removed microneurosurgically. Postoperatively, the patient underwent whole-brain irradiation (30Gy). Due to the advanced stage of the malignancy and to gain control over further intracerebral metastasis, which were not visible on the MRI, no local radiotherapy such as radiosurgery was chosen. No postoperative or radiation-related complications were observed.

**Figure 2:**
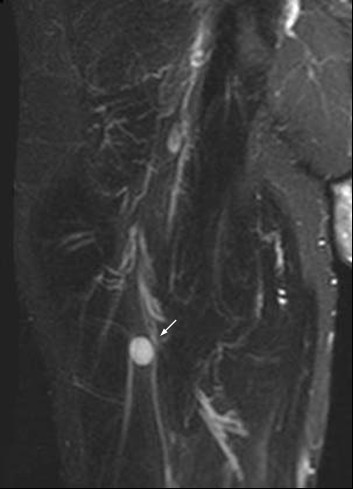
Whole-body MRI (T2WI) identified structure of increased signal intensity in close relation to the sciatic nerve (arrow).

**Figure 3:**
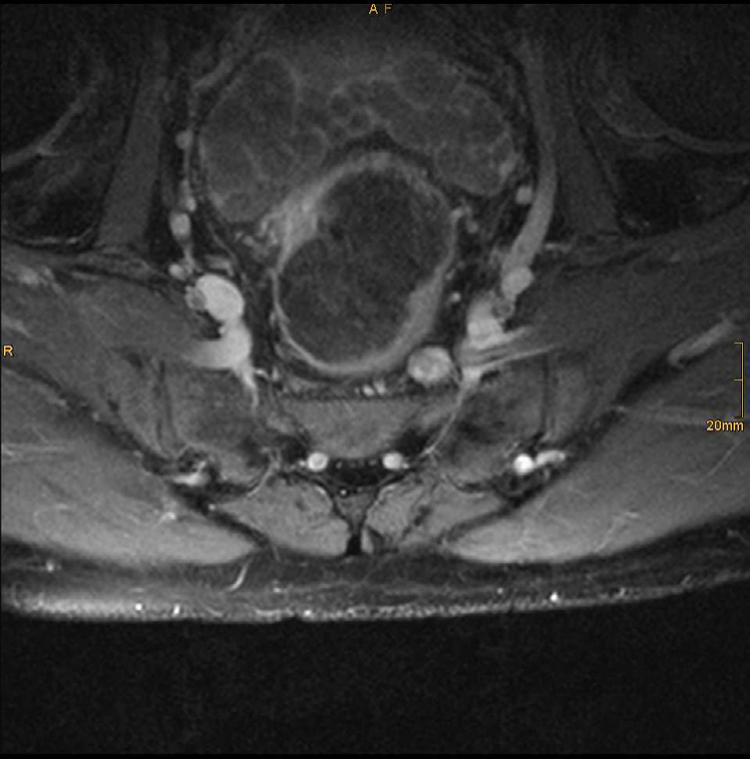
Whole-body MRI (T1WI+Gd) axial sacral slice, identified structure of increased signal intensity in close relation to the right sciatic nerve.

**Figure 4:**
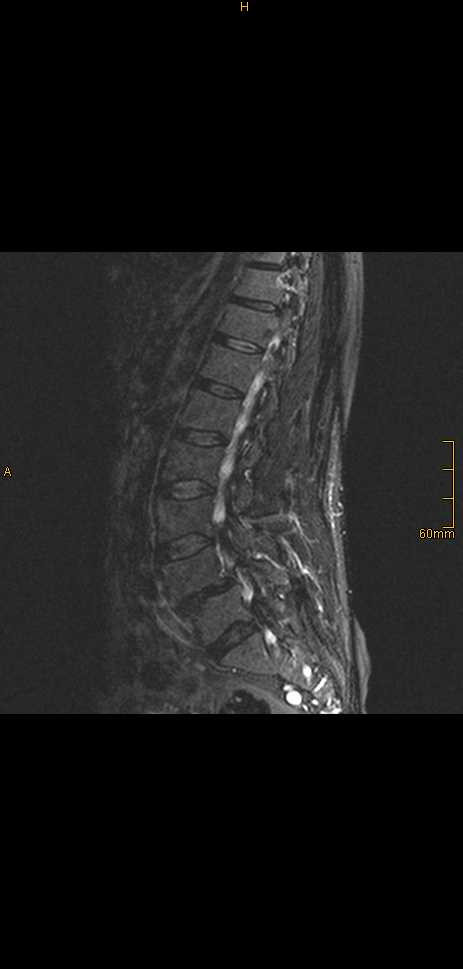
Spinal sagittal MRI (T1WI+Gd) identified tumors in the sacral section of spinal column (S1 and S2).

Fluorodeoxyglycose positron emission tomography (FDG PET) identified no body lesions except for an area of increased accumulation lateral to the right jugular vein, which corresponded to an enlarged lymph node, suggesting a nodal metastasis on MRI. PET even failed to demonstrate the right frontal lesion that was still present prior to cerebral irradiation. Only whole-body MRI (T2WI) identified several structures of increased signal intensity in the right thigh. One of the lesions was contiguous with the sciatic nerve (Figure 2 and 3). The radiologic criteria were indicative of a peripheral nerve tumor, suggesting that this was the primary tumor. Spinal MRI demonstrated further metastases at the level of S1 and S2 but no relationship to spinal nerves (Figure 4).

Based on the patient's poor prognosis due to extensive local tumor spread and the presence of distant cerebral and vertebral metastases and a high risk of neurological deficits associated with a resection of the thigh tumors, a removal was considered unjustified. Stigmata of neurofibromatosis were excluded, as were pulmonary and visceral metastases.

### Postoperative follow-up

The first follow-up examination was performed after 3 months. MRI identified no tumor recurrence in the left frontal lobe and no progression of the lesion on the right (Figure 1B+D). The patient had no clinical or neurological symptoms and suffered no seizures on continuous phenytoin medication (300 mg/day). At the last follow-up 11 months later, MRI showed unchanged cerebral findings and no progression of the lumbar vertebral lesions. However, there was an increase in the size of the cervical lymph node metastasis, and MRI for the first time demonstrated metastases in the area of the right thigh. Nevertheless, the patient continued to be free of neurologic deficits or epileptic seizures and no further therapy was initiated.

### Histology

**Figure 5:**
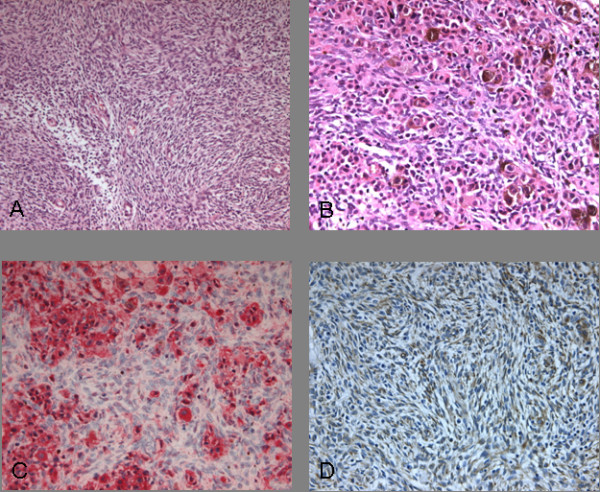
**A **Hematoxylin-Eosin (H&E) staining, with polymorphic, spindled cells in nestlike clusters (10×). **B **Melanin within epitheloid cells (20×). **C **Positive immunreaction against S-100 (20×). **D **Positive immunreaction against VIM (20×).

Hematoxylin-eosin (H&E) staining demonstrated tumor tissue with spindled epithelioid cells, some of which contained melanin (Figure 5A+B). The cells were arranged in nestlike clusters with an altogether heterogeneous appearance. Immunohistochemically, strong expression of vimentin (dilution 1:600; Zymed, Wien/Austria) and S-100 (dilution 1:6000; DAKO, Glostrup/Denmark) was demonstrated (Figure 5C+D). The pigmented cells were positive for HMB45 (dilution 1:500; DAKO, Glostrup/Denmark) and there was isolated expression of EMA (dilution 1:100; DAKO, Glostrup/Denmark). The overall expression of Ki67 antigen (dilution 1:200; DAKO, Glostrup/Denmark) was low, but some foci with an index of 30% were identified. There was no immunoreaction to LU-5 (cytokeratin; dilution 1:500; Biomedicals, Augst/Switzerland), NF (neurofilament; dilution 1:200; Zymed, Wien/Austria), or SYN (synaptophysin; dilution 1:150; BioGenex; San Ramon/U.S.A.). Furthermore electron microscopy investigation showed a basal lamina, strongly indicating peripheral nerve origin. The histological diagnosis of MPNST was based on the demonstration of spindled cells and their arrangement, the pattern of immunoreactions as well as the electron microscopy investigation. Due to the overall low expression of the Ki67 antigen and the histological characterization, the tumor was graded WHO°I, according to the WHO classification for soft-tissue tumors[5]. The histological diagnosis was verified by several reference pathologists.

## Conclusion

The diagnosis of a MPNST is a clinical challenge for the physician regarding treatment options and additional diagnostic steps. In particular, if the tumor has spread into the Central Nervous System. No uniform criteria have been established for the treatment of MPNST outside the central nervous system. However, it is widely agreed that the extent of surgical resection is an important prognostic factor while no definitive data is available on the benefit of postoperative radiotherapy[2]. Irradiation seems to reduce local recurrence but does not affect the overall survival rate. Evidence for any relevant cytotoxic effect of chemotherapy (doxorubicin, ifosfamide) is not available[7]. Chemotherapy is performed only with palliative intent.

In most published cases of MPNST metastasizing to the cerebral parenchyma, the metastasis was removed microsurgically. The majority of reports are from Japan (Table 1). In some cases, surgical resection was followed by whole-brain irradiation. Our case is of interest because the patient had two cerebral metastases of which only one was removed surgically. It is thus possible to compare the outcome after combined surgery and radiotherapy with the outcome after irradiation only. In our case, there has been local tumor control of both metastases for 14 months so far. Therefore we conclude that whole-brain irradiation without prior surgical removal or a radiosurgical approach in treating cerebral metastasis of a MPNST is the best choice.

MPNST in general has an unfavorable prognosis with a 5-year survival rate of only 64%. Survival is even much shorter in patients with a brain metastasis and is not affected by the therapeutic approach. A definitive appraisal of the situation is difficult because only few patients have been treated so far. One patient survived for 18 months without treatment [8] whereas another died a few months after total resection of a brain metastasis[9]. In our case, resection and irradiation have so far suppressed local cerebral recurrence for a total of 14 months while there has been progression of the other distant metastases. Given the rapid tumor progression in our case, cautious therapy with little harm to the patient is indicated.

Beside the clinical challenge of a new diagnose MPNST, the pathologist has to deal with a great work up in the beginning to provide the physician with a correct diagnosis. The histological diagnosis of MPNST requires careful differentiation from a number of tumor entities with similar histologic features. In general, MPNST cells have either an epithelioid or spindled architecture with gradual transitions between the two. The spindled type must be carefully differentiated from other spindled-cell soft tissue tumors, in particular fibrosarcoma. An immunoreaction to S-100 as in our case excludes a fibrosarcoma and underlines the close association of MPNST with the nerve sheaths[9,10]. Another tumor to be considered in the differential diagnosis in our patients was malignant melanoma because some cells were found to contain melanin on microscopic inspection. However, a malignant melanoma could be excluded because there was no immunoreaction to LU-5 (cytokeratin), which is only expressed in some epitheloid variants of melanomas. Whereas vimentin positivity as in this case, occurs also in high malignant melanomas. Nevertheless, evidence of a basal lamina by electron microscopy investigation strongly indicates the tumor as a MPNST by nerve sheath origin. In the case presented here, the tumor was further characterized as a melanocytic MPNST because the melanin-containing cells were found to be positive for HMB45.

## Competing interests

The author(s) declare that they have no competing interests.

## Authors' contributions

J.T.: collected clinical data, wrote particular parts of the paper, performed the reference search

K.M.: histological evaluation, wrote the histology part

G.N.: performed all MRI's, included the figures, wrote the MRI findings

J.L.: responsible for the irradiation of the patient, contribute in the follow-up of the patient and in the revision of the manuscript

J.V.: contributed in data collection, revision and translation of the manuscript, performed surgery

## Pre-publication history

The pre-publication history for this paper can be accessed here:



## Figures and Tables

**Table 1:** Cases of MPNST with brain metastasis

Case	Author	Age/Sex	NF^1)^	Site of primary tumor	Localization of Brain metastasis	Treatment of brain metastasis	Survival time ^2)^
1	D'Agostino, 1963 [11]	22/f	yes	Cytomatic process	Temporal	Not given	Not given
2	White, 1971 [12]	20/m	yes	Lumbar spinal nerve	Medulla oblongata	Not given	Not given
3	Doi, 1983 [13]	23/m	yes	Chest	Multiple	Incomplete resection, irradiation	3 months
4	Hasegawa, 1984 [14]	51/m	yes	Thoracolumbar spinal nerve	Sylvian fissure	None (biopsy only)	3 1/2 months
5	Hirose, 1989 [15]	53/m	no	Paravertebral muscle	Parietal	Resection, irradiation, chemotherapy	Not given
6	D'Angelo, 1991 [16]	68/f	no	Lower arm	Parieto-occipital	Resection	13 months
7	Fenzi, 1995 [8]	45/f	no	Brachial plexus	Parietal	None	18 months
8	Haisa, 1996 [17]	58/f	no	Maxillary sinus	Dura mater	Resection	Days^3)^
9	Oishi, 2000 [18]	48/m	no	Abdomen	Frontal	Resection	Not given
10	Tsuchiya, 2004 [19]	84/f	no	Medial angle of eye	Not given	Not given	Not given
11	Matyja, 2004 [9]	33/m	No	Chest	Multiple	Resection	Months^4)^

^1) ^Type 1 neurofibromatosis

^2) ^After diagnosis of a brain metastasis

^3) ^Postoperative death due to hemoptysis

^4) ^No. of months not specified
